# The Ultrasonic Directional Tidal Breathing Pattern Sensor: Equitable Design Realization Based on Phase Information

**DOI:** 10.3390/s17081853

**Published:** 2017-08-11

**Authors:** Arijit Sinharay, Raj Rakshit, Anwesha Khasnobish, Tapas Chakravarty, Deb Ghosh, Arpan Pal

**Affiliations:** TCS Research and Innovation, Kolkata 700156, India; raj.rakshit@tcs.com (R.R.); deb.ghosh@tcs.com (D.G.); arpan.pal@tcs.com (A.P.)

**Keywords:** ultrasonic phase detection, tidal breathing, 3D printed blow pipe, AD8302, pulmonary assessment

## Abstract

Pulmonary ailments are conventionally diagnosed by spirometry. The complex forceful breathing maneuver as well as the extreme cost of spirometry renders it unsuitable in many situations. This work is aimed to facilitate an emerging direction of tidal breathing-based pulmonary evaluation by designing a novel, equitable, precise and portable device for acquisition and analysis of directional tidal breathing patterns, in real time. The proposed system primarily uses an in-house designed blow pipe, 40-kHz air-coupled ultrasound transreceivers, and a radio frequency (RF) phase-gain integrated circuit (IC). Moreover, in order to achieve high sensitivity in a cost-effective design philosophy, we have exploited the phase measurement technique, instead of selecting the contemporary time-of-flight (TOF) measurement; since application of the TOF principle in tidal breathing assessments requires sub-micro to nanosecond time resolution. This approach, which depends on accurate phase measurement, contributed to enhanced sensitivity using a simple electronics design. The developed system has been calibrated using a standard 3-L calibration syringe. The parameters of this system are validated against a standard spirometer, with maximum percentage error below 16%. Further, the extracted respiratory parameters related to tidal breathing have been found to be comparable with relevant prior works. The error in detecting respiration rate only is 3.9% compared to manual evaluation. These encouraging insights reveal the definite potential of our tidal breathing pattern (TBP) prototype for measuring tidal breathing parameters in order to extend the reach of affordable healthcare in rural regions and developing areas.

## 1. Introduction

Pulmonary diseases affect not only urban but also rural demographics worldwide [1,2,3,4]. Obstructive airway diseases (OAD), viz., chronic obstructive pulmonary disease (COPD) is the second largest cause of death worldwide [1,2]. Abrupt changes in weather, lifestyle, pollution, smoking habits and a dearth of awareness are the main causes of adverse effects in the pulmonary system, triggering respiratory ailments at an epidemic scale [5,6,7]. COPD, pneumothorax, and obstructive sleep apnea (OSA), to name a few, are barely detected at their initial stages [1,2,5]. Since most OADs are not reversible and are thereby never completely curable, it is crucial to detect these ailments at the earliest stage possible. Additionally, it is very difficult to differentiate between the types of OADs, for example COPD is mostly misdiagnosed as asthma [6].

The gold standard for assessment of pulmonary conditions is spirometry [7,8,9,10]. Spirometry measures pulmonary function parameters such as forced vital capacity (FVC ≈ 5.4 L), inspiratory and expiratory reserve volume, forced expiratory volume in 1 s (FEV_1_ ≈ 4.6 L), peak expiratory flow rate (PEF ≈ 10 L/s), maximal voluntary ventilation (2.5–3.3 L) and many others [11,12,13]. In order to compute these lung function parameters, the procedure of spirometry has a stringent requirement for the participant to forcefully breathe in (and out) following a very complex process, which is hard to follow and implement by most participants. Spirometry is not at all recommended for patients who are suffering from severe lung conditions, elderly people and children less than 6 years of age. Opportunely, a new direction in the diagnosis of respiratory diseases is emerging that relies on the analysis of tidal breathing patterns, i.e., natural and undisturbed breathing at a resting state without any intentional forceful effort [13,14,15,16,17,18,19,20,21,22,23,24,25]. Expediently, researchers [13,14,15,16,17,18,19,20,21,22,23,24,25] have disclosed a variety of respiratory parameters that can be derived solely from tidal breathing patterns which are sufficient to distinctly diagnose various lung conditions [18]. It is found that some crucial physiological indications relating to pulmonary mechanics and the respiration control mechanism are deducible from tidal breathing patterns for both normal adults and infants. Moreover, the kind and extent of airway obstruction disease perturbs the tidal breathing pattern signals for both children and adults [13]. The pre-requisite of this line of diagnosis/pulmonary assessment is an affordable, efficient and user friendly device to acquire and analyze the tidal breathing pattern.

A few direct and indirect methods exist to measure tidal breathing parameters [26,27,28]. Flow meters use a direct method to measure the respiratory flow characteristics, including thermal and ultrasonic flow meters [10,29,30,31]. Respiration rates are also measured indirectly using the electrocardiogram (ECG) [32], photoplethysmography (PPG), pulse oximetry [33], tracheal breath sounds [34], impedance pneumography [35] and other methods [36,37]. Tracheal breath sounds measurements are highly susceptible to environmental noises. Piezoelectric respiration transducers [38], pressure mattresses [39], abdominal strain gage sensors [40], microphone [41], camera-based vision analysis techniques [28], and accelerometers [42] have been utilized for measure respiratory rates, volume and flow parameters. Peak flow meters [43] are portable and cheap devices which measure expiratory flow rates, but are not meant for tidal breathing pattern evaluation. Nevertheless, most of these techniques mainly measure the respiratory rate and/or tidal volume, rather than the complete breathing pattern acquisition and evaluation. Additionally, the user interface, the ease of operation, and provision for handling in absence of medical staff are not considered for most of the techniques. Calibration time and effort is another major challenge of most of these emerging techniques as well as the existing pulmonary function testing (PFT) techniques. While there are various ways to measure air-flow, viz., mechanical, pressure, optical, thermal, vortex, electromagnetic, Doppler and ultrasound-based flow meters [29,30], one of the most accurate ones is based on ultrasound-based measurement [29,30,31]. However, most of the ultrasound flow measurement devices utilize a time-of-flight (TOF) principle that makes the instrumentation requirement quite stringent [29,44]. This is because under the TOF measurement principle a sharp pulse is required to be generated, coupled with the requirement of superior time resolution in the detector circuit, particularly when the flow velocity becomes significantly small compared to the sound speed. For example, it only takes 294.12 µs for sound to travel through a 10-cm blow pipe (assuming sound speed = 340 m/s). Tidal breathing velocity, which can be as small as 0.1 m/s [13,25,45], when added to sound speed yields 294.03 µs for the sound pulse traveling the same distance (assuming tidal blow air flows along the sound path). This points towards a 0.09-µs time resolution in the receiver circuit that in turn requires sub-micron pulse generation in the transmitter and a suitable noise reduction scheme on the receiver side as the receiver needs to be wide band for accepting the sharp pulse. In contrast, a phase detection technique based on continuous wave generation is simpler to implement and offers enhanced resolution as it is possible to measure a very small phase difference based on today’s integrated circuits (ICs), leading to measurement of very small flow velocities. Thus the phase detection (PD) technique can simplify the circuit configuration greatly [44,46]. In fact, the contribution in this work lies in instrumenting a very sensitive yet affordable tidal breathing sensor/recorder based on an ultrasound phase detection technique that is later validated through state-of-the art studies.

In an attempt to surmount the demerits of the existing techniques, mentioned above, the current work presents an ultrasound-based, novel, equitable, efficient, user-friendly and portable device to acquire and analyze tidal breathing pattern (TBP) in real time, aimed towards pulmonary condition assessment. This work is aimed at developing a sensor that can acquire and analyze a tidal breathing pattern without the need for conscious and/or forced breathing maneuvers. The complete system is developed using 40-kHz air-coupled piezoelectric ultrasound transreceivers, one radio-frequency (RF) phase-gain IC [47,48], a few opamps and an Atmega micro-controller. In order to achieve high sensitivity, changes in phase measurement techniques of ultrasonic waves have been exploited (in contrast to usual TOF measurements), thus making the sensor highly sensitive so that it can record the directional tidal air flow, even through the nostrils. The measurement of phase difference-based respiration flow and/or velocity measurement accomplished by utilizing phase-gain IC8302 is another novelty achieved here. This sensor is developed in an effort to be employed as a screening device for pulmonary assessment, not as a replacement of spirometry. We have, in recent past, demonstrated the feasibility of designing a tidal breathing apparatus using the phase detection method [49]. In this work, we present a detailed study on the sensor design where we optimized and calibrated for angled ultrasound towards accurate detection of tidal breathing. Moreover, we also conducted a detailed analysis of breath cycles to extract tidal breathing parameters. The developed system is calibrated using a standard 3-L calibration syringe (Medikro syringe 3000 [50]) and is also parametrically compared with a standard spirometer [51]. It is to be noted that we collected data from healthy subjects only and have carried out first-level validation for healthy subjects compared with reported state-of-art studies containing clinical assessment.

The measurement principle is outlined in Section 2, followed by a description of the system architecture in Section 3. Tidal flow estimation employing the present developed sensor is outlined in Section 4. Preprocessing of the acquired tidal breathing pattern (TBP) signal and computation of respiratory parameters are depicted in Section 5. Section 6 presents details of the experiments and discusses the computed results. The final section outlines the concluding remarks.

## 2. Measurement Principle

We employed an ultrasound-based phase detection principle for accurately detecting tidal breathing patterns. For a given distance between the ultrasound transmitter (Tx) and receiver (Rx), the change in sound speed due to inspiration or expiration results in a proportionate phase change between the transmitted and received signal.

Mathematically, if *t* is the time taken by the ultrasound signal to travel a distance *D* between the transducers, then phase-difference, φ, is given by,

(1) $$\varphi =\frac{2\pi t}{T}=\frac{2\pi }{T}\left(\frac{D}{v_{sound}+v_{P}}\right)$$

where *T* is the periodicity of ultrasound excitation, $v_{sound}$ is the velocity of sound and $v_{P}$ is the velocity component of the medium (say, due to respiration) parallel to sound travel path. Hence, the change in the phase difference ∂φ can be related to change in the respiration velocity ∂v as,

(2) $$\partial \varphi =-\frac{2\pi \;D}{T}\frac{\partial v_{p}}{{\left(v_{sound}+v_{p}\right)}^{2}}=K\;\partial v_{p}$$

Since speed of sound is much greater than tidal breathing component (≈ 0.1 to 10 m/s for tidal breathing), we can write (without any loss of generality) ${\left(v_{sound}+v_{P}\right)}^{2}\approx v_{sound}^{2}$, thus resulting in $K=-\frac{2\pi D}{Tv_{sound}^{2}}$.

Equation (2) clearly shows that the component of inspiration/expiration velocity along the sound travel path, $\partial v_{P}$, is directly proportional to phase shift (∂φ) of the ultrasound wave. This also reveals that the sensitivity is directly proportional to ultrasound frequency since for a given flow velocity, a larger phase shift is obtained for increasing frequency (f = 1/T). However, we opted to operate at 40 kHz as air-coupled transducers for this frequency are widely available and are cost effective.

## 3. System Architecture

**Figure 1:**
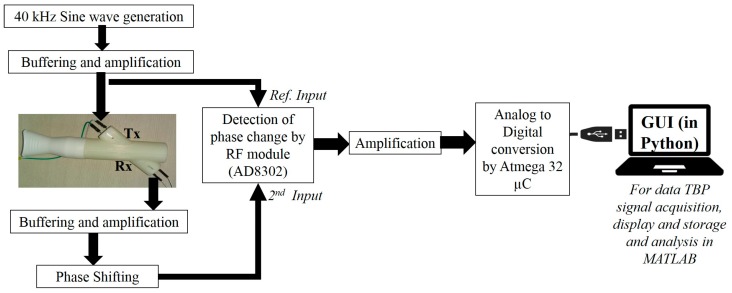
The entire system architecture comprising the flow pipe, the electronic circuitry and acquisition in laptop through Python-based bespoke software GUI. TBP: tidal breathing pattern.

This section presents the complete sensor architecture comprising a ‘3D printed’ blow pipe (fitted with ultrasound Tx and Rx), phase detection electronics, and a PC/laptop running data acquisition and visualization graphical user interface (GUI). This is illustrated in Figure 1. Each component is described in the following sub-sections.

### 3.1. The 3D printed Flow Pipe

**Figure 2:**
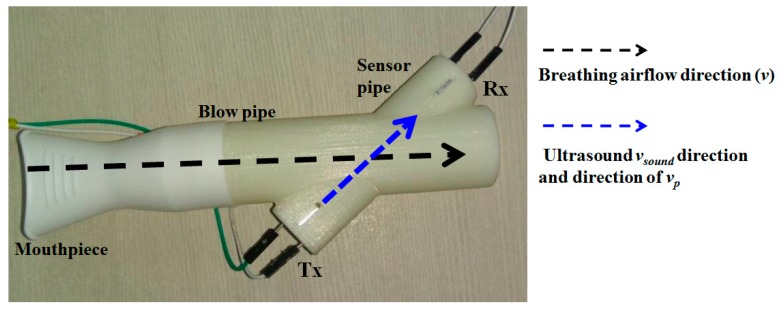
The 3D printed flow pipe.

Figure 2 displays the flow-pipe which is designed in-house and is 3D printed. It consists of a main cylindrical hollow pipe fitted with a disposable mouth-piece for inhaling and exhaling. Ultrasound air-coupled transducers (one receiver and one transmitter) are kept at an angle (*θ* = 40°) with respect to the main blow pipe 68 mm apart, and hosted inside another hollow cylinder (sensor pipe), as depicted in Figure 2. The angle is kept at 40° to ensure maximum dynamic range coverage. The justification is given in Section 4.3.

### 3.2. The Electronic Circuitry

The entire device is USB-powered and uses a DC–DC converter to power the electronic boards. The 40-kHz air-coupled ultrasonic Tx is driven by a 40-kHz sine wave (generated using ICL8038) followed by a variable gain buffer amplifier. The ultrasonic Rx is similarly connected to a buffer and a variable gain amplifier followed by an active phase shifter module. This phase shifter module is used to adjust the idle (or no flow condition) operating point so that the default phase shift (due the Tx to Rx path lengths) is compensated for. The phase shifter output is fed to AD8302 (Analog Devices, Norwood, MA, USA)—the phase detector module which compares the received signal with the reference excitation signal. AD8302 (Analog Devices) is a phase-gain measurement IC targeted for operation in high-frequency regions (typically from the MHz to GHz range). It takes two inputs in pin INA and pin INB and provides gain (INA/INB) and phase (phase difference between INA and INB) information in terms of proportionate DC voltage (scaled between 0 to 1.8 V). The output from AD8302 is finally amplified (scaled between 0 to 5 V) and fed to the analog to digital converter (ADC) of an Atmega32 microcontroller (µC) which digitizes the data at 100 Hz sampling rate and sends it to a laptop/computer through USB communication.

### 3.3. GUI

**Figure 3:**
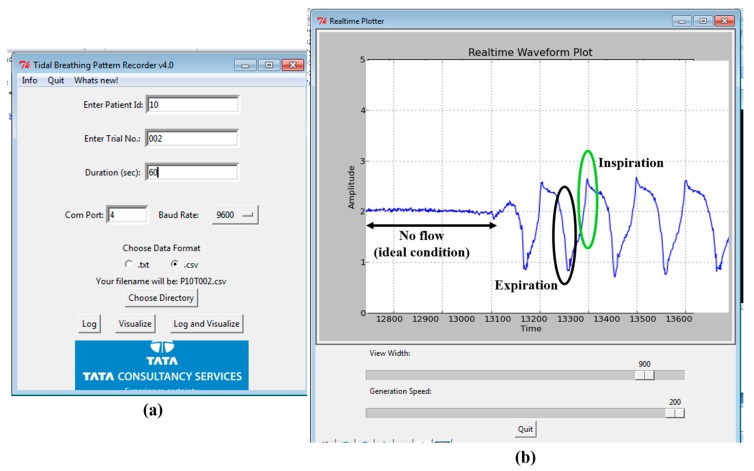
The custom-made TBP acquisition software interface (**a**) the initial screen to enter the subject and trial information; (**b**) the data visualization window depicting the acquired TBP waveform.

A user-friendly software interface is developed in Python. This has the provision to visualize and save the recorded data in systematic manner along with recording patient metadata (age, height and weight, trial number, trial duration) while keeping the anonymity intact. Figure 3a shows the opening page of the GUI to collect user information (meta-data) while Figure 3b shows the visualization screen. An experimenter operates the GUI to acquire the TBP signal; at the end of the data acquisition, the interface displays the computed tidal breathing parameters (extracted TBP parameters are explained in Section 5.2). The GUI is easy to operate without any medical supervision. The experimenter is needed to control the phase shifter to set the baseline at the start of the data acquisition. We now focus on actual tidal flow calculations based on our system design and the measurement principle as described above.

## 4. Tidal Flow Estimation Based on Actual Configuration

This section describes the derivation of tidal velocity, flow and volume computations and parametric evaluations when configured with angled beam ultrasound and measured with the AD8302 phase/gain IC. The concluding part of this section explains the selection of *θ* for the angled beam ultrasound.

### 4.1. Tidal Velocity, Flow and Volume Computations

**Figure 4:**
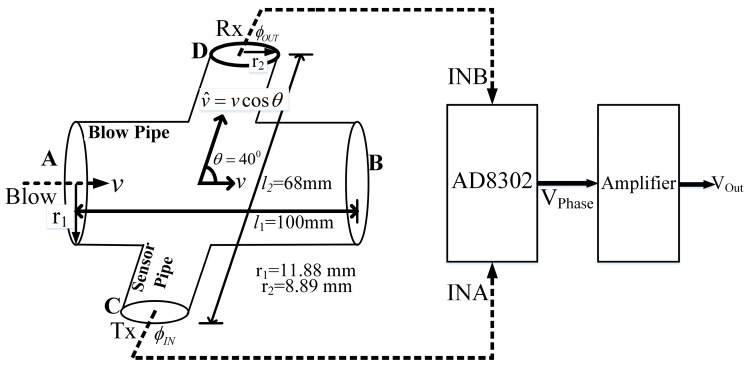
The flow pipe arrangement for phase detection to comprehend respiration flow.

The schematic view and the dimensions of the flow pipe is depicted in Figure 4 and it is assumed that the air flow inside the pipe is laminar.

We have the main blowpipe with a constant radius of *r*_1_ = 11.88 mm, throughout the length. The component of respiration velocity that lies in the ultrasound path (C–D) is given by,

(3) $$v_{P}=v\;\cos\theta$$

where, v is the respiration velocity applied by the subject at point A (Figure 4) and *θ* is the angle between the two cylindrical pipes. Hence, the change in the phase difference ∂φ is related to the change in the respiration velocity, $v_{P}$, as in Equation (2) and can be rewritten according to the blowpipe specifics as,

(4) $$\partial \varphi =-\frac{2\pi l_{2}}{Tv_{sound}^{2}}\partial v_{P}$$

where, $l_{2}$ is the distance between the ultrasonic Tx and Rx in the sensor pipe. The Tx and Rx signals are then fed to first input (INA) and second input (INB) pins of the AD8032 module, and the output voltage $V_{Phase}$ from AD8302 is given as (from datasheet) [48],

(5) $$V_{Phase}=K_{\varphi }\left[\varphi_{INA}-\varphi_{INB}\right]$$

where, $K_{\varphi }$ is the phase gradient given as mV/rad and $\varphi_{INA}$/$\varphi_{INB}$ are the phase values associated with the Tx and Rx signals, respectively. Next, $V_{Phase}$ is amplified with a variable amplification factor *G*. Hence the output at the final stage, $V_{OUT}$ can be written as,

(6) $$\begin{array}{l} \partial V_{OUT}=GK_{\varphi }\left[\varphi_{INA}-\varphi_{INB}\right]=GK_{\varphi }\partial \varphi =-\frac{GK_{\varphi }2\pi l_{2}}{Tv_{sound}^{2}}\partial v_{P}\\ \Rightarrow \partial V_{OUT}=-\frac{GK_{\varphi }2\pi l_{2}\cos\theta }{Tv_{sound}^{2}}\partial v \end{array}$$

where, the negative sign indicates that the direction of air flow and the output voltage have opposite polarity. Thus the respiration velocity, ∂v, can be represented as,

(7) $$\partial v=-\frac{Tv_{sound}^{2}}{GK_{\varphi }2\pi l_{2}\cos\theta }\partial V_{OUT}=F\left(G,K_{\varphi }\right)\;\partial V_{OUT}$$

Here, the term $F(G,K_{\varphi })$ indicates the dependence of the output voltage change (for a given air flow) on the overall gain of the system as well as the phase gradient $K_{\varphi }$, other parameters being constant. We can compute the theoretical value for $F(G,K_{\varphi })$. However, this needs to be estimated (through the calibration process) in the present setup in order to obtain respiration velocity in terms of m/s.

Now, the instantaneous flow rate, *Q* (m^3^/s) for laminar flow is given as,

(8) $$Q=\oint_{A}v\cdot dA=v\pi \;r_{1}^{2}$$

where v is the respiration velocity obtained from Equation (7), and *A* is the cross-section area. From the two equations, namely Equations (7) and (8), we obtain,

(9) $$Q=F\left(G,K_{\varphi }\right)\;V_{OUT}\pi r_{1}^{2}\cdot 10^{3}\;(\mathrm{in}\;L/s)$$

### 4.2. Parametric Evaluation

The value of G and $K_{\varphi }$ needs to be computed in order to derive *Q*. In order to get $K_{\varphi }$, the module AD8302 needs to be tested at our operating frequency of 40 kHz. The AD8302 datasheet [37,38,39,40,41,42,43,44,45,46,47,48] presents a linear phase response in the range 0° to 180°. The datasheet indicates that the IC can uniquely measure a phase shift only from 0° to 180°, after which it wraps the phase.

**Figure 5:**
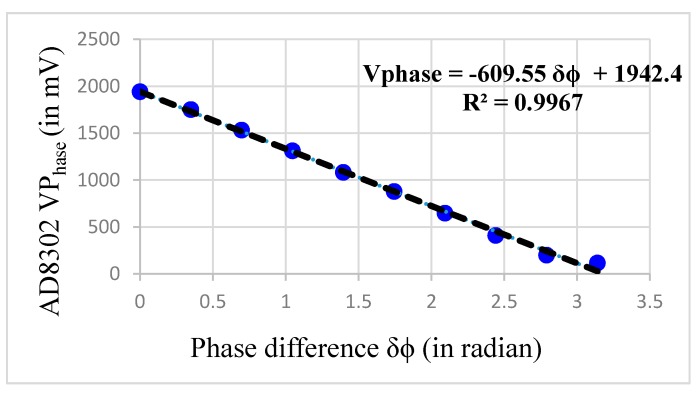
The change in *V_Phase_* with change in phase difference at 40 kHz.

Figure 5 shows the phase response curve when 40-kHz sine wave (1 V peak-to-peak) is fed at INA and is set as the reference signal. The same signal with a variable phase shift (0° to 180°) is fed to INB. It is observed that the response is fairly linear in 0° to 180° range. However, it is also identified that from 160° onwards, the response starts to saturate. Therefore, we limited our measurements range between 0° and 160° only. The slope of the curve, i.e., *K_ϕ_*, is found to be −609.55 mV/rad. In our design we kept the operating point for idle condition (i.e. no flow condition) at 90 degrees (i.e., middle of the curve) so as to capture excursions (i.e., inhale and exhale) on both sides without any phase wrapping or saturation. Effectively, we reserved a 60° downside swing for exhale and 60° degree upward swing for inhale (this reduction of 20 degree works out fine as even with forced breathing the phase-shift remained well under 60 degree). This working scheme based on the measured phase response curve further influenced our decisions to select the ultrasound beam near the 40° angle (i.e., *θ*) to the main blow pipe as described in the next subsection.

The term $F\left(G,K_{\varphi }\right)$ relies on the system gain *G* and the slope $K_{\varphi }$. Now, $K_{\varphi }$ is computed to be −609.55 mV/rad and is treated as fixed. The gain (*G*) of the variable gain amplifier is nominally set to 3, in an effort to achieve maximum compliance during data acquisition. Since $F\left(G,K_{\varphi }\right)$ now depends only on the variable gain *G*; we write $F\left(G,K_{\varphi }\right)=F(G)$, here onwards.

In Equation (7), by substituting the values of T (= 25 µs), $v_{sound}$ (= 347 m/s), when the temperature is 27 °C, $r_{1}$ (= 0.01188 m), $K_{\varphi }$ (= −609.55 mV/rad), $l_{2}$ (= 0.068 m), θ (= 40°) and G (= 3), we obtain the theoretical value of F(G)=5.11. However, the exact value of F(G) may differ from its theoretical i.e., the ideal value. In order to estimate the exact value of F(G), the TBP system needs to be calibrated. The system calibration is outlined in Section 6.1.

### 4.3. Selecting the Optimal Angle (θ)

The maximum velocity of human tidal breathing rarely exceeds 3 m/s [13,45]. In order to attain maximum compliance of the tidal breathing pattern recorder (TBPR) device i.e., to accommodate voluntary forceful breathing also, the maximum flow velocity is considered to be 10 m/s which is then used for computation of optimal angle *θ*. In the previous section, we have mentioned that the working region of TBPR is taken from 0° to 160°. We have also set the idle operating point at 90°. Putting $v=v_{\max}$ in Equations (3) and (4), we find the magnitude of maximum change in phase as,

(10) $${\left|\partial \varphi \right|}_{\max}=\frac{2\pi l_{2}v_{\max}}{T\;v_{sound}^{2}}\cos\theta$$

**Figure 6:**
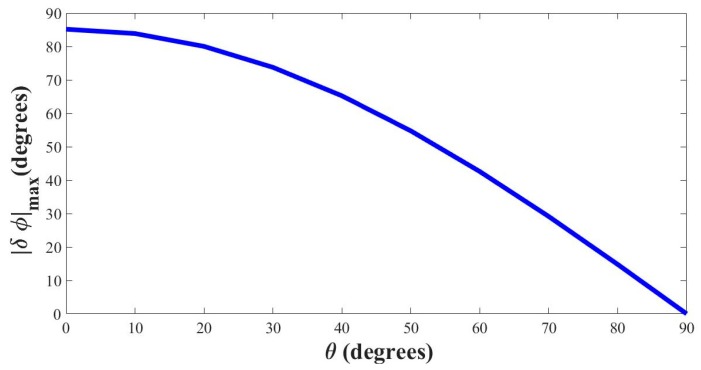
Change in phase $\partial \varphi_{\max}$ with respect to the angle *θ* (*v*_max_ = 10 m/s).

Figure 6 shows the variation of ${\left|\partial \varphi \right|}_{\max}$ over varying values of *θ* considering $v_{\max}=10\;m/s$.

From Figure 6 it is seen that with flow velocity set at 10 m/s the device loses sensitivity when *θ* is nearing 90°, thereby requiring much higher effort by the subjects (beyond the realm of tidal breathing) to attain substantial change in phase. At *θ* = 90° the phase does not change at all with injected blow. In contrast, for low values of *θ*, the phase change approaches 90° which is more than maximum allowable phase swing that was set to 60° previously. In addition, setting *θ* = 0° is impossible from a design perspective as it would make the blow and sensor pipe lie on the same line. Also, low values of *θ* increase the length of the sensor pipe unnecessarily. We found *θ* value of 40° to be quite workable as it offers a very compact design for the sensor pipe, and the corresponding phase change falls around 60° both ways. Hence, the phase swing with this particular choice of *θ* stays within 30° to 150° around the no-blow condition (90°) for expiration and inhalation, respectively.

## 5. Tidal Breathing Analysis

This section describes the basic preprocessing steps for extracting tidal breathing parameters. The acquired tidal breathing (TBP) signal is analyzed in a MATLAB 2016 environment in order to extract significant respiratory traits, essential for assessing the tidal breathing parameters.

### 5.1. Preprocessing

**Figure 7:**
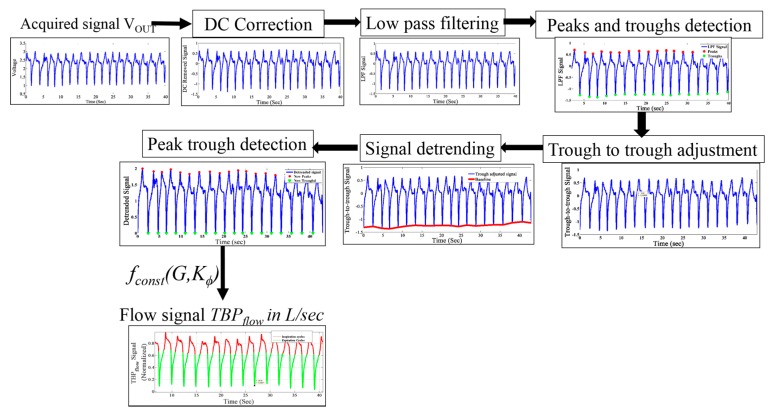
The TBP signal preprocessing steps.

The acquired signal is in voltage $V_{OUT}$ from which the respiration flow rate (in L/s) is obtained according to Equation (9). The preprocessing steps are illustrated in Figure 7. The mean value of the signal is then subtracted from $V_{OUT}$ itself to obtain the DC-corrected signal representing the change in the blow injected by the subject. The TBP signal is contaminated with powerline interferences, body movement and other high frequency noises. To eliminate these types of contamination, a second order infinite impulse response (IIR) low pass filter with a cut-off frequency 15 Hz is applied on the DC-removed signal.

Peaks and troughs of the TBP signal, which are crucial elements of inspiration-expiration cycles, are detected on the low pass filtered signal. The peak–trough detection algorithm identifies the local maxima and minima in the low pass filtered signal.

Using the locations of the troughs in the signal, the signal is then trough-to-trough-adjusted (i.e., the signal starts and ends with a trough) for better subsequent window detection. This is achieved at the cost of a losing a few data points, which will affect the already tracked peak and trough locations.

Using cubic interpolation, the non-linear drifts in the signal are removed, without compromising the peak-trough correspondence, considering the breath-by-breath TBP flow signal of a 5-s window.

Subsequently, the respiratory parameters are computed from the pre-processed signal.

### 5.2. Tidal Breathing Signal Parameters

**Figure 8:**
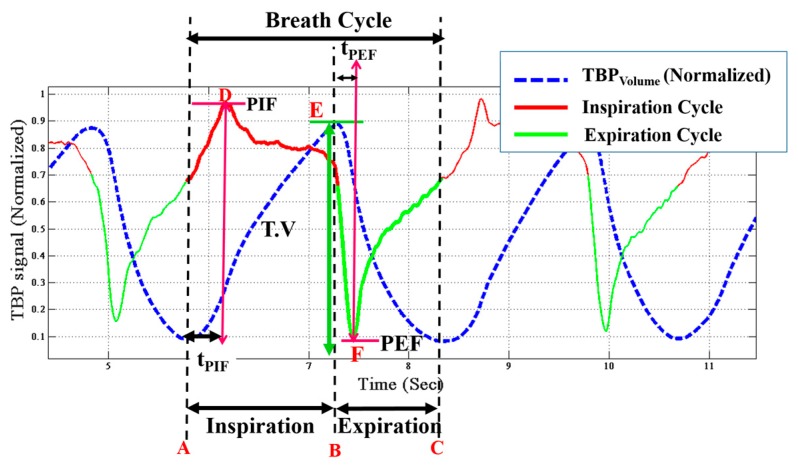
Tidal breathing pattern signal with respiratory parameters annotated. PIF: peak inspiratory flow; PEF: peak expiratory flow; t_PEF_: Time to peak expiratory flow; t_PIF_: peak inspiratory flow.

Tidal breathing contains principal comprehension associated with pulmonary system, its condition, its working and control mechanisms [13,17,18,19,21,23,25]. This information is attained not only from the flow pattern but also using volumetric means. The TBP signal, acquired using our device, directly conveys the flow (TBP_flow_) signal. Thus the first step, prior to extracting the essential parameters, is to obtain the volumetric signal (TBP_volume_). The TBP_flow_ signal is integrated with respect to time (using trapezoidal rule of numerical integration) to obtain TBP_volume_. Figure 8 shows the preprocessed TBP_flow_ as well as the derived TBP_volume_ (blue dotted line) signals. It is to be noted that by convention [13,17,19,21], the inspiratory cycle is shown as positive and vice versa for the expiratory cycle. However, in our system, the *V_Phase_* has a negative slope, which when substituted in Equation (6) turns $F\left(G,K_{\varphi }\right)$ positive. Thus in our system, exhalation leads to a positive voltage swing. However, for representation, we have reversed the cycle as per standard literature [7,8,13,17,19,21].

To derive the respiratory parameters, it is essential to detect the onset and end of inspirations and expirations [15]. The inspiration cycle is from the minima to the maxima while the expiration cycle is from the maxima to the next minima of the same TBP_volume_. Corresponding to Figure 8, A–C is a breathing cycle, where A–B is the inspiratory cycle and B–C is the expiratory cycle. A number of independent and dependent attributes of respiratory signals are computed from the TBP_volume_ and TBP_flow_ signals. These parameters [13,17,18,19,21,23,25] are described below with reference to Figure 8:

- Inspiratory time (TI) is the time elapsed from onset (A) to the end (B) of inspiration. It is given in seconds.
- Expiratory time (TE) is the time taken to expire, i.e., the time elapsed between the onset (B) to the end (C) of expiration.
- Breathing rate (BR) is the number of breaths cycles per minute (BPM) is computed by the following equation,
  (11) $$BPM=\frac{60}{TI+TE}$$
- Duty cycle (DCy) is given by the equation,
  (12) Duty Cycle=TI/(TI+TE)
- Peak inspiratory flow (PIF) is the maximal flow rate attained during every inspiratory cycle. It is obtained from the TBP_flow_ signal. In Figure 8 the point D corresponds to PIF.
- Peak expiratory flow (PEF) is the minima in the TBP_flow_ signal during every expiratory cycle, i.e., the maximal flow attained during expiration. It refers to point F in the Figure 8.
- Time to peak inspiratory flow (t_PIF_) is the time taken to reach the maximum flow rate during inspiration from its onset, i.e., the time taken to reach D from A.
- Time to peak expiratory flow (t_PEF_) is the time elapsed from to onset of expiration (E) till PEF is attained.
- Tidal volume (T.V) is represented in terms of inspiratory and expiratory tidal volume. Inspiratory (TV_ins_) and expiratory tidal volume (TV_exp_) are the total volume of air inspired and exhaled respectively. TV_ins_ refers to the area under the curve between point A and B and TV_exp_ is that between the point B and C in the TBP_flow_ signal. TV_ins_ is the same as the tidal volume (T.V). During restful tidal breathing is the TV_ins_ (i.e., T.V) is around 500 mL, however, it can vary largely during stimulated tidal breathing [52], as in this current study.
- Inspiratory (*v*_ins_) and expiratory (*v*_exp_) velocity are the velocities during inhalation and exhalation, respectively.

## 6. Experiments and Results

This section presents the system calibration, the experimental setup for tidal breathing data logging and the computed results. Finally the present work is compared with relevant prior works.

### 6.1. System Calibration

**Figure 9:**
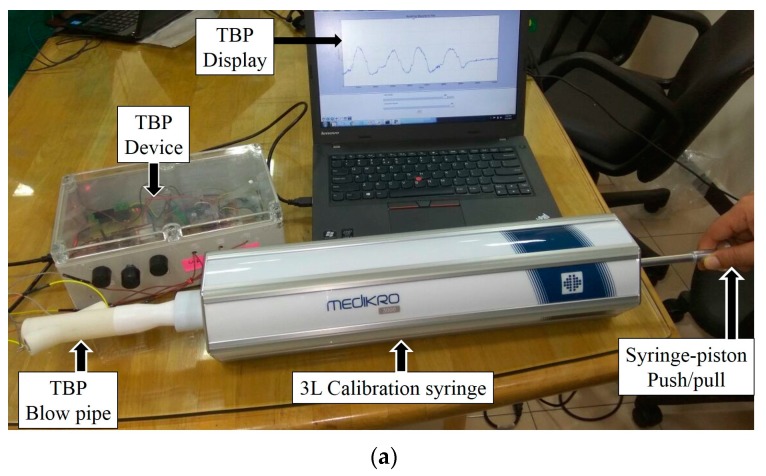

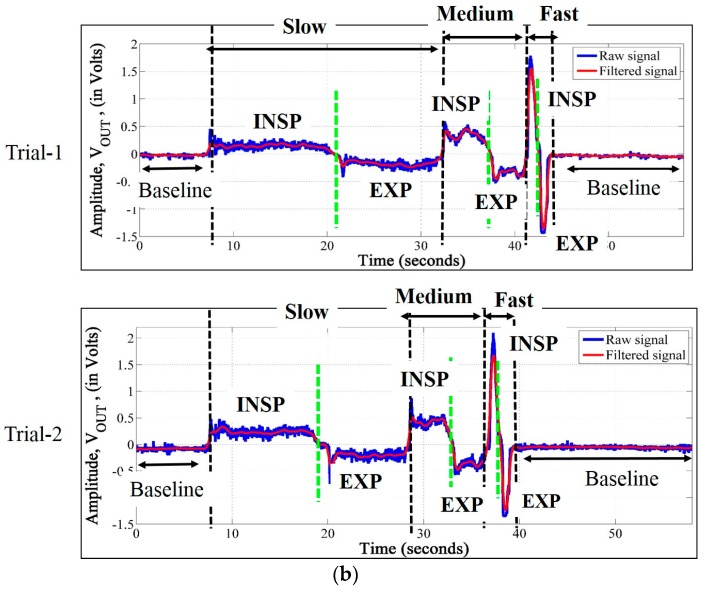
(**a**) The setup comprising a 3-L syringe (fitted at the mouthpiece of the blowpipe) in series with a TBP sensor during the calibration phase; (**b**) variation in the output of TBP device (*V_OUT_*) during slow, medium and fast rate of syringe-piston push (EXP expiration) and pull (INSP: inspiration).

Our system is calibrated using standard 3-L syringe (Medikro 3000 [50]), used for calibrating spirometers, for various ranges of flow rates. The TBP sensor developed by us relies only on tidal breathing acquisition and analysis. It is known that during tidal breathing, the tidal volume varies around 300 mL–2 L [52]. The calibration setup is depicted in Figure 9a. The TBPR blow pipe is attached to the calibration syringe and the complete setup is kept at rest on a steady horizontal platform. The piston of the syringe is pulled out (≈inspiration) and pushed in (≈expiration) manually at three different speeds (slow, medium and fast) i.e., varying the average flow rate. We have carried out three trials of syringe expirations and inspirations for each of the three flow rates. Figure 9b visually illustrates the variation in the output voltage (*V_OUT_*) of our TBP device for the three different flow rates. Table 1 presents the area under the curve (AUC) (averaged over three trials), in volt-seconds, obtained by integrating the *V_OUT_* signal during expiration (EXP) and inspiration (INSP) for all the slow, medium and fast flow rates. It can be observed from Table 1 that there exists minimal difference between the AUC during inspiration and that of expiration, the maximum of which is found out to be 160 mL.

The acquired TBP signal, *V_OUT_*, is first filtered to eliminate high frequency noises and then utilized to compute the calibrated *F*(*G*) by integrating Equation (9), which can be written as,

(13) $$\begin{array}{l} \int_{t_{1}}^{t_{2}}Q(t)\;dt=\int_{t_{1}}^{t_{2}}F_{meas}(G)\;\pi \;r_{1}^{2}\;10^{3}V_{OUT}(t)\;dt\\ \Rightarrow vol=F_{meas}(G)\;\pi \;r_{1}^{2}\;10^{3}\int_{t_{1}}^{t_{2}}V_{OUT}(t)\;dt\\ \Rightarrow F_{meas}(G)=\frac{vol}{\pi \;r_{1}^{2}\;10^{3}AUC} \end{array}$$

where, vol = 3 L (as a standard 3 L syringe is used for calibration), *r*_1_ = 0.01188 m, $AUC=\int_{t_{1}}^{t_{2}}V_{OUT}(t)\;dt$ in volt-seconds, *t*_1_ is the start time of inspiration (expiration) and *t*_2_ is the end time of inspiration (expiration) in seconds, and $F_{meas}(G)$ is the measured value of *F*(*G*). With the total volume of the calibration syringe being constant at 3 L, we have computed the average flow rates during slow, medium and fast syringe piston push/pull to be 0.19 L/s, 0.72 L/s and 2.1 L/s respectively, which are within the tidal breathing range. Table 2 presents $F_{meas}(G)$ during inspiration and expiration for these three different flow rates. The results are averaged over three trials for each of the flow-rates. The mean of the $F_{meas}(G)$ during inspiration and expiration is found out to be ${\hat{F}}_{meas}(G)$ = 5.54. Thus, Equation (9) can be rewritten as,

(14) $$Q={\hat{F}}_{meas}(G)\;V_{OUT}\;\pi \;r_{1}^{2}\;10^{3}\;(L/s)$$

The rest of the signal analysis is based on this calibrated system output according to the Equation (14).

### 6.2. System Validation using Standard Spirometer

**Figure 10:**
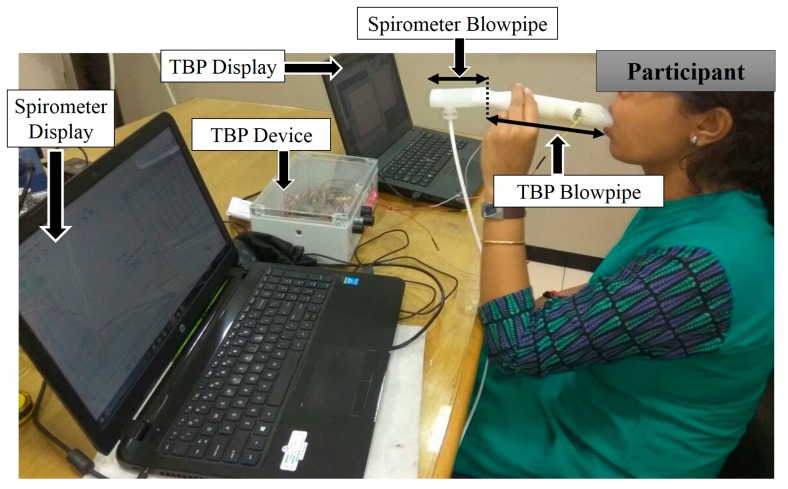
Subject breathes through the blowpipes of TBP and spirometer (connected in series) during the system validation phase.

Our TBP system prototype is validated with respect to a standard spirometer, the Medikro Nano Spirometer [51]. Five participants (2 female, 3 male, age 30 ± 5 years, with no history of pulmonary disease) were asked to sit in a chair with an arm rest and back rest during the experimental session of system validation. Our TBP blowpipe and the Medikro Nano-Spirometer blowpipe were attached in a series, as shown in Figure 10, in a manner such that there was no leakage of air at the device junction. The subjects held the blow pipe in one hand, while their nostrils were closed using a nose-clip, and breathed for 10 s through the blowpipes (connected in series) such that the TBP device and the spirometer acquires the same breath-signals almost synchronously. The subjects are instructed to breathe normally without any forceful effort. Since most of the parameters obtained by a standard spirometer are related to forceful breathing, we have considered two parameters, namely, PEF and PIF, for the comparison with our device. Table 3, shows PIF and PEF as computed by our TBPR prototype and the standard spirometer. It can be observed that the obtained values from TBP device is in tandem with the standard spirometer while the percentage error is less than 16%. These encouraging insights reveal the definite potential of our TBP prototype for measuring tidal breathing parameters.

### 6.3. TBP Data Acquisition

The complete tidal breathing analysis is done on data from healthy subjects from our laboratory. Twenty participants in the age range of 23–51 years, consisting of 6 females and 14 males participated in this experiment. All the experimental procedure are Helsinki declaration [53]-compliant and ethical clearance was sought from the Tata Consultancy Services (TCS) institutional review board (Ref. No. TCS_IRB/ESD_TBPR, dated 27 April 2017).

The subjects were made to sit on a comfortable chair with back and arm rests. The objective and procedures of the experiment were explained to them. Subjects were instructed to tidally breathe in and blow out without any voluntary effort for 60 s. Three such trials, one trial each day for three consecutive days, were taken for each subject. During this procedure, the nose was closed using a nose clip, so that entire inhalation and exhalation occurred through the mouth. The closing of nose with a nose clip is a common practice followed during lung function tests through spirometry. However, it has been noted that whenever a subject is asked to consciously respire through the pipe, the respiration gets stimulated causing increase in respiratory rate than the natural restful tidal breathing. This has been reported to be a normal psychological response in prior works [52]. However, the advantage of the present technique is that it does not require the participants to respire forcefully and to hold breaths.

### 6.4. Experimental Results and Discussion

Tidal breathing pattern is acquired by employing the developed system and analyzed to extract vital respiratory attributes. The computed respiratory attributes are evaluated with respect to already reported values in state-of the-art works.

#### 6.4.1. Computing Tidal Breathing Parameters

Instead of calculating only the breathing rate and/or tidal volume, evaluating a set of respiratory parameters can provide more insight towards pulmonary condition monitoring [13,17,18,19,21]. Although we were not performing any screenings of pulmonary artifacts in this present work, we extracted some vital respiratory parameters from the TBP_flow_ and TBP_volume_ as shown in Table 4. For the listed parameters, we indicated the average value of the three trials conducted for each of the subjects, along with the standard deviation given in parenthesis. The standard deviation for all parameters are minimal, indicating that the intra-subject variance over the number of trials is very small. This also confirms the repeatability of the TBP device. Ideally, the inspiratory (VT_ins_) and expiratory (VT_exp_) tidal volume should be very close, if not equal, which is readily verified from the results in Table 1.

As per the physiology of restful tidal breathing, expiration time (TE) is slightly more than that of inspiratory time (TI) [52]. In the present experimental setup where the subjects are actively conscious about the ongoing data recording and where the blow pipe is used for breathing, the act of tidal breathing is not restful. That is why TE is less than TI for all the subjects. Moreover, as the total lung capacity for each of the subjects is fixed, even with an honest attempt to perform tidal breathing, *v*_exp_ is greater than *v*_ins_. This is in conjunction with the observed fact that TE < TI keeps the fixed lungs capacity theory intact. As *v*_exp_ > *v*_ins_, PEF is also higher than PIF, this validates the entire hypothesis of subjects’ conscious and stimulated breathing.

**Figure 11:**
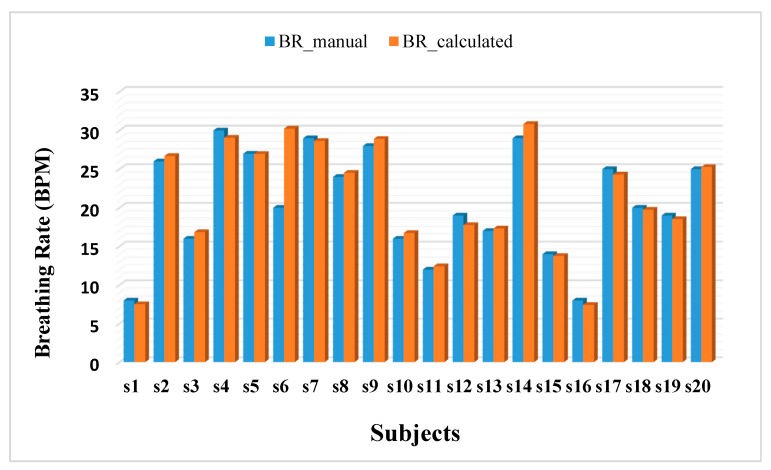
Breathing rates computed by the experimenter (BR_manual) and by calculating the TI and TE (BR_calculated) from the TBP signal.

Figure 11 depicts the breathing rates (breaths per minute, BPM), of each subject computed from the TBP signal (BR_calculated) and counted manually by the experimenter (BR_manual). Both these parameters are represented as averaged over all the trials per subject. It is observed that the percentage error over all the subjects is 3.39%. This implies the correctness of the TBP device.

**Figure 12:**
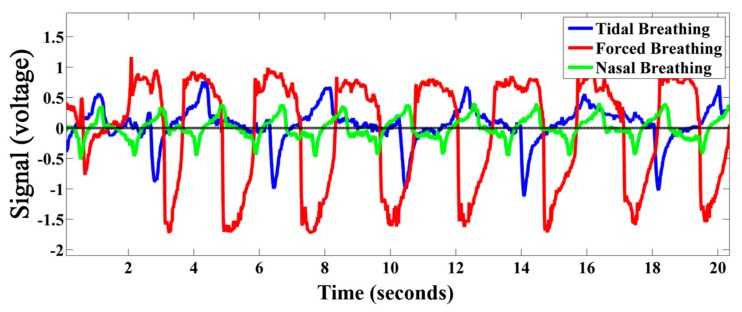
Tidal, forced, and nasal breathing acquired by the developed TBP device.

In addition to tidal breathing, the developed TBP device is capable of acquiring forceful breathing without any saturation as well as very low intensity nasal breathing (breathing only through nose). This is depicted in Figure 12. Thus the developed device can work over a wide operating range. Although the current work do not provide any analysis of forceful and nasal breathing, the device can also be employed for acquiring these types of breathing if required.

#### 6.4.2. Comparison with Existing Relevant Works

As restful tidal breathing gets stimulated by conscious effort and/or by introduction of any mask of pipe in mouth/nose, researchers [14,16,17,18,19,20,21,22,24] dealing with tidal breathing analysis have acquired and analyzed data mostly from infants and/or children. The tidal breathing attributes of infants/children vary largely from those of the adult population. Hence, for comparing the current work we have chosen only the most relevant literature that are dealing with the adult population only. Table 5 indicates the parameters evaluated by each method (methods are indicated in column 1). For a particular method, if a specific attribute has not been computed then that column is left blank (‘-’). The number of subjects (N) participated in this study is also comparable and is relevant for performing reasonable analysis. It is observed that all the parameters are reasonably comparable with the prior arts [13,23,52,54,55,56]. Moreover, our outcome variance is also very small; therefore, the developed TBP sensor can be efficiently implemented for evaluating respiratory indicators from tidal breathing.

## 7. Conclusions

We have presented the design, parametric evaluation, system calibration and experimental results of the developed novel ultrasonic directional TBP sensor. We have judiciously utilized the ultrasonic phase detection technique to build a novel, equitable, efficient, user-friendly, portable yet highly sensitive solution for pulmonary health monitoring. The developed sensor facilitates acquisition of continuous directional tidal respiration airflow that can be utilized to extract typical tidal breathing parameters. In fact, the particular configuration covers a wide dynamic range, in the sense that it can acquire very feeble air flow from tidal-nasal expiration to voluntary expirations through the mouth. The TBP device has been systematically calibrated against a standard 3-L calibration-syringe for various flow ranges and has also been parametrically compared with standard spirometer with a percentage error of less than 16%. In addition, our tidal breathing analysis showed ample resemblance with the reported tidal breathing parameters for healthy subjects. This immediately opens up the horizon for exploiting the recent trend of tidal breathing analysis for assessing one’s pulmonary health conditions in contrast to painful spirometry tests. However, even at the current stage, our effort shows sufficient sensitivity and accuracy to pose the devised sensor prototype (based on our design approach) as an affordable tidal breathing analyzer/recorder for application in pulmonary assessment, at least in the preliminary screening stages before one is prescribed for more rigorous and specific pulmonary function tests like spirometry. We also plan to test our system on subjects with unhealthy respiratory systems and to extend the systematic signal analysis of the acquired TBP signals.

## Figures and Tables

**Table 1:** Area under the curve (AUC) (in volt-seconds) calculated from variation in the output voltage (*V_OUT_*) signal for slow, medium and fast inspirations and expirations using the 3-L calibration-syringe. EXP: expiration; INSP: inspiration.

Flow Rates	Slow		Medium		Fast	
	INSP	EXP	INSP	EXP	INSP	EXP
AUC	1.48	1.32	1.23	1.11	1.17	1.09

**Table 2:** ${\hat{F}}_{meas}(G)$ computation by analyzing *V_OUT_*.

Flow Rate	${F_{meas}(G)|}_{Insp}$ during Inspiration	${F_{meas}(G)|}_{Exp}$ during Expiration
Slow	4.57	5.12
Medium	5.51	6.09
High	5.78	6.20
Mean	5.29	5.80

**Table 3:** Comparison of PIF and PEF computation with a spirometer and our TBP system.

	PEF (L/s)			PIF (L/s)		
Subjects	Spirometer	TBPR	% Error	Spirometer	TBPR	% Error
**1**	0.97	1.1	13.40	1.13	1.3	15.04
**2**	0.8	0.67	16.25	0.71	0.6	15.49
**3**	2.21	2.2	0.45	1.7	1.5	11.76
**4**	1.85	2.13	15.13	1.57	1.7	8.28
**5**	1.78	2.01	12.92	1.7	1.5	11.76

**Table 4:** Attributes computed from TBP_flow_ and TBP_volume_ for all twenty subjects. BR: breathing rate; TI: inspiration time; TE: expiration time; DCy: duty cycle; BPM: breaths cycles per minute; VT_ins_: inspiratory tidal volume; VT_exp_: expiratory tidal volume.

Sub	BR (BPM)	TI (s)	TE (s)	DCy	PIF (L/s)	t_PIF_ (s)	PEF (L/s)	t_PEF_ (s)	VT_ins_ (L)	VT_exp_ (L)	*v*_INSP_ (m/s)	*v*_EXP_ (m/s)
1	7.51 (0.72)	3.93 (0.19)	3.59 (0.20)	0.49 (0.03)	1.03 (0.05)	1.38 (0.15)	1.60 (0.16)	0.85 (0.09)	2.75 (0.27)	2.59 (0.16)	2.33 (0.11)	3.49 (0.12)
2	26.7 (1.11)	1.19 (0.17)	1.07 (0.08)	0.51 (0.04)	0.82 (0.04)	0.53 (0.02)	0.98 (0.16)	0.58 (0.16)	0.61 (0.07)	0.64 (0.10)	1.84 (0.11)	2.21 (0.31)
3	16.84 (1.01)	1.83 (0.03)	1.68 (0.15)	0.50 (0.04)	1.41 (0.01)	0.92 (0.05)	1.73 (0.03)	0.65 (0.02)	1.70 (0.03)	1.82 (0.05)	3.19 (0.01)	4.1 (0.04)
4	29.06 (0.91)	1.04 (0.11)	0.98 (0.06)	0.51 (0.03)	0.92 (0.05)	0.52 (0.03)	1.04 (0.01)	0.47 (0.01)	0.63 (0.01)	0.64 (0.01)	2.08 (0.13)	2.35 (0.01)
5	26.96 (1.5)	1.06 (0.08)	1.01 (0.05)	0.51 (0.01)	1.7 (0.05)	0.53 (0.06)	1.37 (0.01)	0.48 (0.01)	0.88 (0.04)	0.89 (0.04)	3.12 (0.13)	3.86 (0.01)
6	30.24 (0.87)	1.04 (0.06)	0.97 (0.02)	0.51 (0.01)	1.06 (0.01)	0.52 (0.01)	1.21 (0.01)	0.47 (0.01)	0.72 (0.04)	0.74 (0.03)	2.75 (0.01)	3.31 (0.06)
7	28.64 (2.77)	1.18 (0.08)	1.10 (0.08)	0.52 (0.01)	1.38 (0.04)	0.58 (0.02)	1.57 (0.06)	0.53 (0.01)	1.06 (0.04)	1.11 (0.02)	3.11 (0.11)	3.59 (0.13)
8	24.51 (3.9)	1.57 (0.23)	1.37 (0.15)	0.53 (0.01)	1.44 (0.01)	0.71 (0.12)	1.75 (0.06)	0.65 (0.04)	1.49 (0.13)	1.45 (0.10)	3.26 (0.04)	3.98 (0.15)
9	28.90 (1.95)	1.15 (0.13)	1.05 (0.05)	0.52 (0.02)	1.25 (0.03)	0.58 (0.01)	1.47 (0.09)	0.50 (0.01)	0.95 (0.05)	0.99 (0.10)	2.82 (0.07)	3.34 (0.29)
10	16.73 (0.34)	2..02 (0.02)	1.66 (0.08)	0.56 (0.02)	1.63 (0.07)	0.88 (0.06)	2.27 (0.04)	0.69 (0.01)	2.26 (0.04)	2.23 (0.04)	3.82 (0.16)	5.12 (0.09)
11	12.42 (0.61)	2.82 (0.21)	1.92 (0.12)	0.56 (0.04)	1.43 (0.03)	1.03 (0.27)	2.07 (0.13)	0.89 (0.10)	2.10 (0.11)	2.13 (0.02)	3.23 (0.04)	4.68 (0.03)
12	17.76 (1.05)	1.81 (0.10)	1.53 (0.07)	0.52 (0.02)	1.34 (0.17)	0.70 (0.02)	1.56 (0.01)	0.86 (0.16)	1.48 (0.04)	1.32 (0.17)	3.02 (0.38)	3.54 (0.21)
13	17.31 (0.41)	1.89 (0.14)	1.60 (0.07)	0.54 (0.02)	1.46 (0.05)	0.94 (0.11)	1.94 (0.06)	0.66 (0.05)	1.80 (0.09)	1.90 (0.11)	3.29 (0.11)	4.58 (0.11)
14	30.84 (1.58)	0.97 (0.15)	0.93 (0.06)	0.51 (0.01)	0.96 (0.16)	0.49 (0.53)	1.06 (0.27)	0.43 (1.09)	0.60 (0.03)	0.62 (0.02)	2.17 (0.27)	2.34 (0.49)
15	13.76 (0.88)	2.46 (0.17)	1.86 (0.16)	0.54 (0.01)	1.37 (0.05)	1.01 (0.20)	1.99 (0.22)	0.80 (0.01)	2.15 (0.21)	2.22 (0.27)	3.09 (0.12)	4.51 (0.51)
16	7.42 (0.64)	4.15 (0.36)	3.47 (0.21)	0.57 (0.01)	1.29 (0.04)	1.26 (0.21)	2.12 (0.06)	0.83 (0.02)	3.30 (0.05)	3.39 (0.01)	2.92 (0.06)	5.08 (0.04)
17	24.30 (3.12)	1.42 (0.21)	1.40 (0.18)	0.50 (0.03)	1.65 (0.06)	0.75 (0.08)	1.73 (0.07)	0.59 (0.02)	1.54 (0.05)	1.60 (0.05)	3.73 (0.12)	3.99 (0.16)
18	19.75 (0.42)	1.64 (0.04)	1.36 (0.01)	0.56 (0.01)	1.12 (0.09)	0.76 (0.07)	1.60 (0.34)	0.64 (0.02)	1.21 (0.11)	1.37 (0.16)	2.54 (0.21)	3.67 (0.76)
19	18.53 (0.89)	1.82 (0.03)	1.48 (0.03)	0.55 (0.02)	1.32 (0.07)	0.75 (0.09)	1.79 (0.03)	0.66 (0.01)	1.66 (0.05)	1.65 (0.01)	2.97 (0.15)	4.23 (0.04)
20	25.26 (1.62)	1.29 (0.05)	1.18 (0.06)	0.51 (0.02)	1.22 (0.13)	0.68 (0.04)	1.35 (0.19)	0.53 (0.01)	0.03 (0.11)	1.01 (0.09)	2..74 (0.18)	3.05 (0.38)

**Table 5:** Comparison with relevant literature.

Approaches	BR (BPM)	TI (s)	TE (s)	DCy	PIF (L/s)	t_PIF_ (s)	PEF (L/s)	t_PEF_ (s)	VT_ins_ (L)	VT_exp_ (L)	*v*_ins_ (m/s)	*v*_exp_ (m/s)
TBPR	7–30	1.81 ± 0.18	1.56 ± 0.17	0.53 ± 0.02	1.27 ± 0.23	0.77 ± 0.41	1.62 ± 0.32	0.69 ± 0.14	1.49 ± 0.74	1.51 ± 0.74	2.86 ± 0.52	3.76 ± 0.68
[13] *N* = 24	-	1.9 ± 0.5	2.7 ± 0.7	0.41 ± 0.2	0.83 ± 0.4	0.8 ± 0.2	0.64 ± 0.2	1 ± 0.4	0.98 ± 0.18		-	-
[52] (review)	6 to 31	-	-	-	-	-	-	-	0.45–1.6		-	-
[54] (simulation)	-	-	-	-	-	-	-	-	-		0.79–3.16	-
[23] *N* = 16	14 ± 0.4	1.82 ± 0.13	2.37 ± 0.12	-	-	-	-	-	0.77 ± 0.11		-	-
[55] *N* = 15	11–35	-	-	-	-	-	-	-	0.3–3		-	-
[56] *N* = 20	-	-	-	-	-	-	-	-	-			4.7 1.4 (nasal)
